# Spatiotemporal evolution of COVID-19 infection and detection within night light networks: comparative analysis of USA and China

**DOI:** 10.1007/s41109-020-00345-4

**Published:** 2021-02-11

**Authors:** Christopher Small, Daniel Sousa

**Affiliations:** 1 Lamont Doherty Earth Observatory, Columbia University, Palisades, NY 10964 USA; 2 Jet Propulsion Laboratory, California Institute of Technology, Pasadena, CA 91109 USA

**Keywords:** Night light, Spatial network, COVID-19, Spatiotemporal, Connectivity

## Abstract

The spatial distribution of population affects disease transmission, especially when shelter in place orders restrict mobility for a large fraction of the population. The spatial network structure of settlements therefore imposes a fundamental constraint on the spatial distribution of the population through which a communicable disease can spread. In this analysis we use the spatial network structure of lighted development as a proxy for the distribution of ambient population to compare the spatiotemporal evolution of COVID-19 confirmed cases in the USA and China. The Visible Infrared Imaging Radiometer Suite (VIIRS) Day/Night Band sensor on the NASA/NOAA Suomi satellite has been imaging night light at ~ 700 m resolution globally since 2012. Comparisons with sub-kilometer resolution census observations in different countries across different levels of development indicate that night light luminance scales with population density over ~ 3 orders of magnitude. However, VIIRS’ constant ~ 700 m resolution can provide a more detailed representation of population distribution in peri-urban and rural areas where aggregated census blocks lack comparable spatial detail. By varying the low luminance threshold of VIIRS-derived night light, we depict spatial networks of lighted development of varying degrees of connectivity within which populations are distributed. The resulting size distributions of spatial network components (connected clusters of nodes) vary with degree of connectivity, but maintain consistent scaling over a wide range (5 × to 10 × in area & number) of network sizes. At continental scales, spatial network rank-size distributions obtained from VIIRS night light brightness are well-described by power laws with exponents near −2 (slopes near −1) for a wide range of low luminance thresholds. The largest components (10^4^ to 10^5^ km^2^) represent spatially contiguous agglomerations of urban, suburban and periurban development, while the smallest components represent isolated rural settlements. Projecting county and city-level numbers of confirmed cases of COVID-19 for the USA and China (respectively) onto the corresponding spatial networks of lighted development allows the spatiotemporal evolution of the epidemic (infection and detection) to be quantified as propagation within networks of varying connectivity. Results for China show rapid nucleation and diffusion in January 2020 followed by rapid decreases in new cases in February. While most of the largest cities in China showed new confirmed cases approaching zero before the end of February, most of these cities also showed distinct second waves of cases in March or April. Whereas new cases in Wuhan did not approach zero until mid-March, as of December 2020 it has not yet experienced a second wave of cases. In contrast, the results for the USA show a wide range of trajectories, with an abrupt transition from slow increases in confirmed cases in a small number of network components in January and February, to rapid geographic dispersion to a larger number of components shortly before mobility reductions occurred in March. Results indicate that while most of the upper tail of the network had been exposed by the end of March, the lower tail of the component size distribution has only shown steep increases since mid-June.

## Introduction

The spatial distribution of ambient population affects disease transmission, especially when shelter in place orders restrict mobility for a large fraction of the population. At regional scales, the spatial network structure of settlements therefore imposes a fundavmental constraint on the spatial distribution of the population through which a communicable disease can spread. However, traditional population estimation methods aggregate population counts into census blocks of widely varying size, resulting in a progressive loss of spatial detail as population density decreases away from high density urban cores. With confirmed cases of COVID-19 largely reported at USA county levels, the loss of spatial detail is increased further as counties are generally much larger than census blocks. Model simulations using administrative boundaries (e.g. https://www.nytimes.com/interactive/2020/03/20/us/coronavirus-model-us-outbreak.html) illustrate the disparity in county size within the US, and the tendency for larger counties to contain widely dispersed settlements within large areas of effectively uninhabited land area. As populations are dispersed over large numbers of small settlements over most of the peri-urban and rural USA, it is clear that people are not distributed uniformly within counties, and that the administrative boundaries within which data are aggregated are generally irrelevant to disease transmission. A more spatially explicit boundary condition of population and settlement distribution could provide a much more accurate spatiotemporal representation of transmission pathways – particularly when mobility is sharply reduced and transmission occurs within networks of less mobile populations. In this analysis, we investigate the potential influence of the spatial structure of networks of population and development on the transmission of COVID-19 in China and the USA.

Satellite observations of stable night light provide a unique proxy for anthropogenic development in which continuous spatial variations in luminance correspond to intensity of development as density of lighted infrastructure. Brightness and spatial extent of emitted light are correlated with population density (Sutton et al. 2001), built area density (Elvidge et al. 2007) and economic activity (Doll et al. 2006; Henderson et al. 2009) at global scales and within specific countries. The Visible Infrared Imaging Radiometer Suite (VIIRS) Day/Night Band (DNB) sensor on the NASA/NOAA Suomi satellite has been imaging night light at ~ 700 m resolution globally since 2012. The sensor detects both intermittent sources (e.g. fires) and temporally stable light (e.g. settlements). Analysis of higher spatial resolution night light imagery indicates that most of the stable light signal in settlements is from outdoor lighting of infrastructure and transportation corridors (Kyba et al. 2015). Comparisons with sub-kilometer-resolution census observations in different countries across different levels of development indicate that night light luminance scales with population density over ~ 3 orders of magnitude (Small and Barrozo 2020). While night light cannot directly detect intra-urban distributions of population at the highest densities, VIIRS’ constant ~ 700 m resolution can provide a more detailed proxy for population distribution in peri-urban and rural areas where aggregated census blocks lack spatial detail.

In this analysis we use the spatial network structure of lighted development as a proxy for the distribution of ambient population to compare the spatiotemporal evolution of COVID-19 confirmed cases in the USA and China. We quantify this network structure in terms of the rank-size distributions of spatially contiguous lighted areas. By varying the low luminance threshold of VIIRS-derived night light brightness, it is possible to depict spatial networks of varying degrees of connectivity within which population are distributed. Spatiotemporal analysis of confirmed cases at the county (USA) and city (China) levels of aggregation provides a characterization of the temporal trajectories of daily COVID-19 cases in a geospatial context. Projecting the aggregated COVID-19 case counts onto the spatial network structure reveals the degree to which network structure and connectivity may have influenced transmission before and after mobility restrictions were imposed in the USA and China.

## Data

The Visible Infrared Imaging Radiometer Suite (VIIRS) sensor has been imaging night lights from the NASA/NOAA NPP Suomi satellite since 2012. The digital data have been used to produce annual global composites of temporally stable night-time lights from 2015 (Elvidge et al. 2017). Cloud-free annual composites of the night-time visible band VIIRS dnb data provide average brightness in units of nW/(cm^2^ sr). Additional procedures are used to remove ephemeral lights (mostly fires and aurora) and background noise to produce gridded stable lights products. The data and documentation are available from: https://payneinstitute.mines.edu/eog-2/viirs/.

**Fig. 1:**
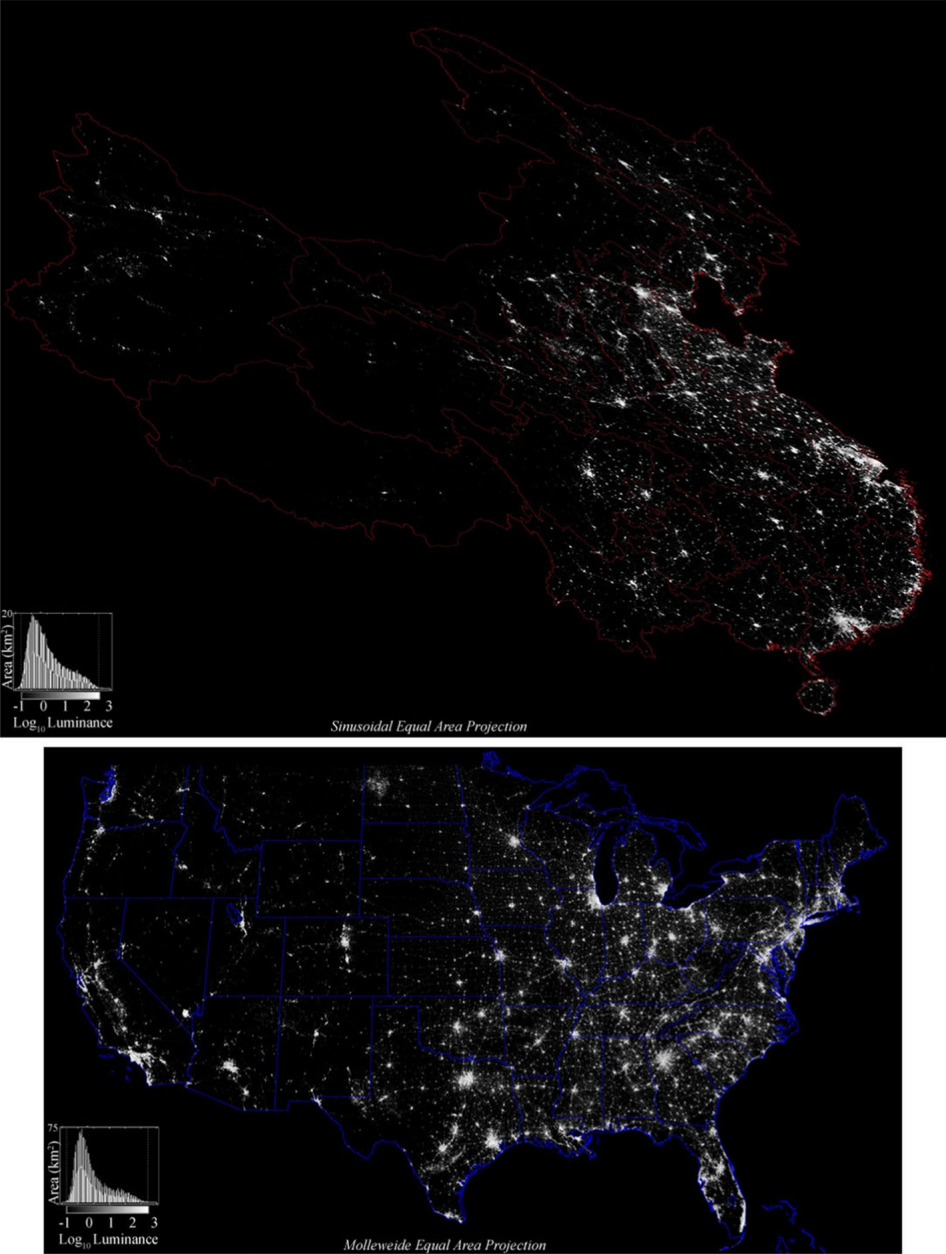
China and conterminous USA night light luminance as imaged by VIIRS in 2015. Because smaller isolated light sources are dimmer, the majority of lights cannot be seen at the scale of this figure. However, network structure is apparent

The VIIRS night light luminance is gridded at 0.004° (~ 500 m at the Equator), oversampling the 700 m native resolution of the VIIRS sensor. We reproject the geographic grids to Molleweide (USA) and Sinusoidal (China) equal area grids with a spatial resolution of 500 × 500 m. Because luminance varies over orders of magnitude, we give results as the Log_10_ of nW/(cm^2^ sr). The reprojected luminance grids are shown, along with the luminance distributions, in Fig. 1. We limit the analysis to the conterminous USA because the lighted areas in Alaska and Hawaii are limited to small numbers of geographically isolated components and are physically separated from the main spatial network within the conterminous USA.

**Fig. 2:**
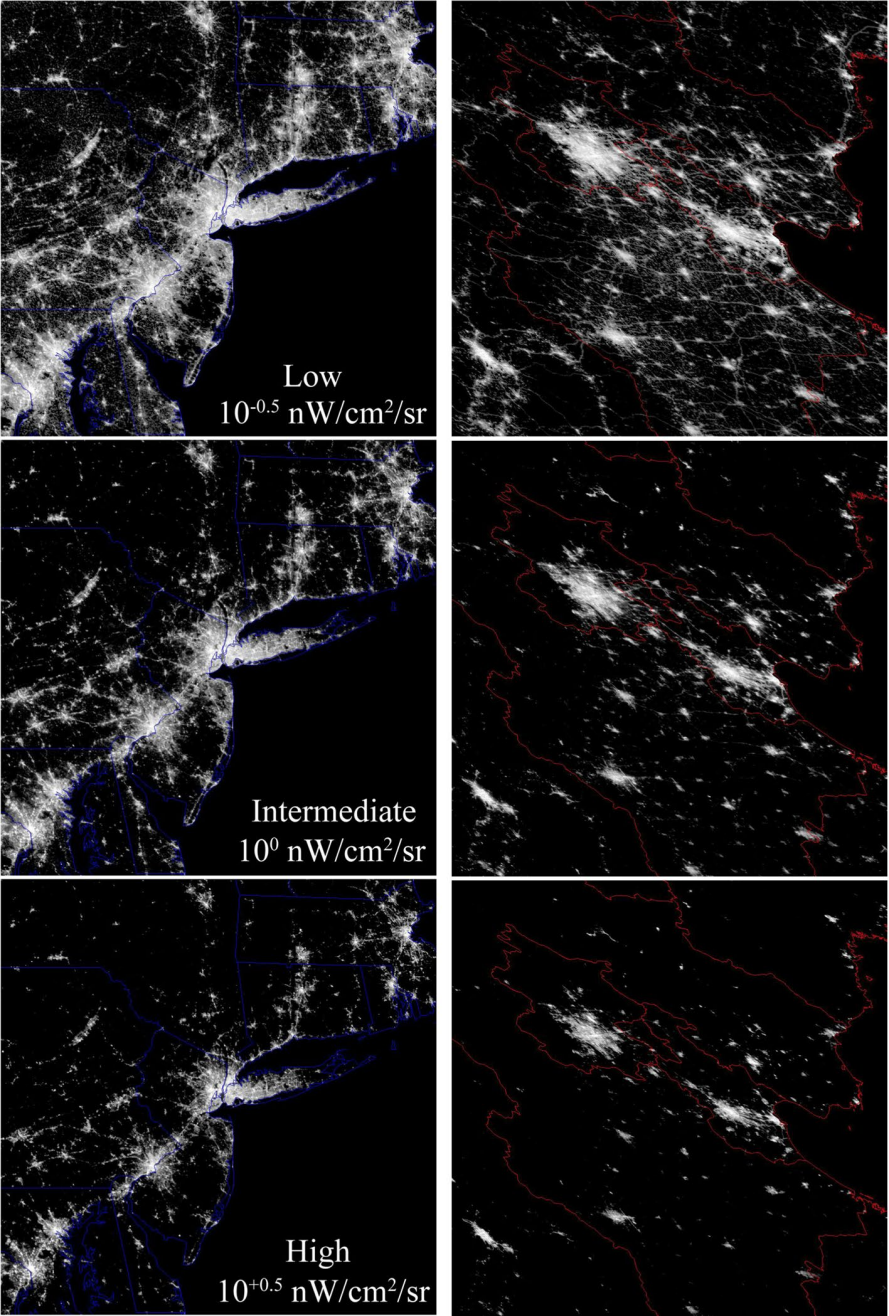
Full resolution comparison of the Northeast Corridor and the Beijing-Tianjin Corridor with three low luminance thresholds. Increasing the threshold attenuates dimmer light pixels at the periphery of network components. The result is to reduce the number, size and connectivity of all components in the network. Luminance scale same as Fig. 1. Enlarge to see full resolution detail

A detailed comparison between the 2015 VIIRS product and 10 m resolution Sentinel 2 optical imagery reveals a systematic relationship between the density of built surface (roads & buildings) and night light brightness (Small 2019). Because different luminance levels in the VIIRS composite generally correspond to different building and road density, different luminance thresholds encompass different types of development. Higher thresholds limit spatial extent to the brightest urban cores with the highest spatial density of outdoor lighting, while lower thresholds also encompass lower density periurban areas where lighting is more intermittent with dimmer, more diffuse sources. For this reason, different luminance thresholds can be considered representative of different ranges of development density, with lower thresholds encompassing a wider range of built environments with greater spatial connectivity. Given the differing implications of different luminance thresholds, it makes sense to treat the threshold itself as a variable in the analysis. In this study, three low luminance thresholds are used to produce three spatial networks of varying degrees of spatial connectivity. The lower threshold (10^−0.5^ nW/cm^2^/sr) attenuates only the lower tail of dimmest pixels, while the upper threshold (10^+0.5^ nW/cm^2^/sr) cuts the primary mode of the distribution and retains only the shoulder and upper tail of brighter pixels. The intermediate threshold (10^0^ nW/cm^2^/sr) retains approximately half of the lighted pixels. Given the late hour (~ 1 AM) of VIIRS’ overpass, the luminance imaged represents a conservative estimate of urban extent resulting from lighted outdoor infrastructure (primarily roads), as studies have shown that many early evening light sources are turned off by midnight.

(Dobler et al. 2015; Li et al. 2020). Figure 2 shows full resolution comparisons of the USA’s Northeast Corridor and China’s Beijing-Tianjin corridor for each threshold. The reduction in size, number and connectivity of network components with increasing threshold is more apparent at full resolution than at the reduced resolution of Fig. 1.

**Fig. 3:**
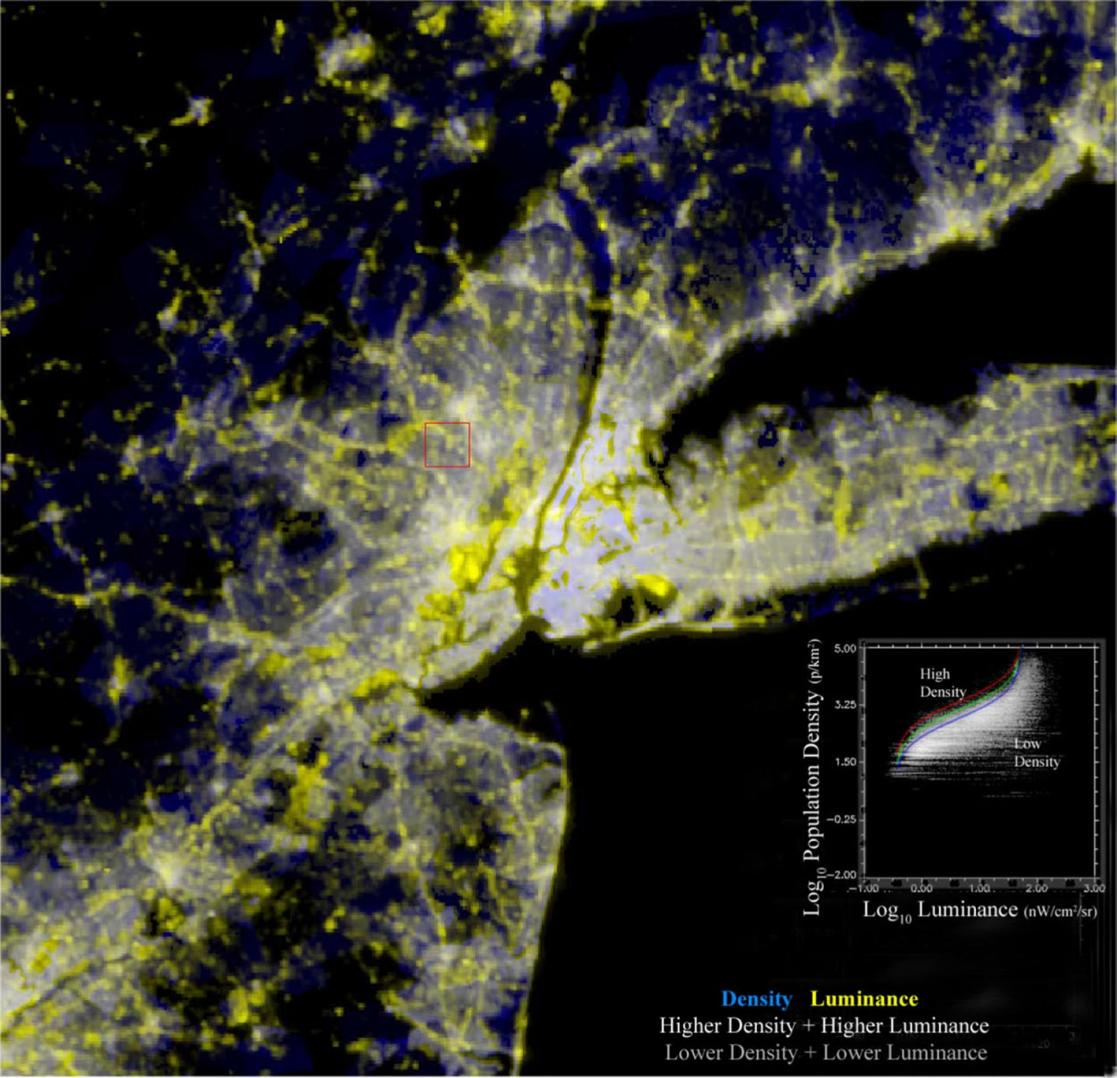
VIIRS night light and population density in the New York Metro area and central Northeast Corridor. Small, high density census blocks approach the 700 m resolution of the VIIRS sensor so the scaling is clearly resolved on the high density tail. Blue rural areas correspond to larger, lower density census blocks extending into unlighted forest and farmland. Yellow in urban areas corresponds to brightly lighted commercial corridors, industrial areas, seaports and airports. The diffuse low density tail of the bivariate distribution is a result of larger census blocks and overglow

The spatial correspondence between night light luminance and residential population density is illustrated by superimposing the 500 m VIIRS luminance and 2010 census blocks of varying spatial resolution (gridded at 500 m). Figure 3 shows the strong correspondence in density and luminance as shades of gray and the disparities in blue and yellow. The larger blue rural census blocks are seen to be mostly unlighted, illustrating the drastic improvement in representation given by night light product over the aggregated census block mapping. The disproportionately luminant areas appear yellow, corresponding generally to commercial corridors (along major thoroughfares), central business districts (e.g. Midtown Manhattan) and industrial areas (NJ Meadowlands) as well as seaports and airports. The inset bivariate distribution in Fig. 3 shows the correspondence between density and luminance spanning at least two orders of magnitude in each dimension. Superimposed on the high density upper tail of the distribution is a suite of offset Logit curves to illustrate the consistent relationship between luminance and maximum density. The more diffuse lower tail of the distribution is less well-defined because of the combined effect of aggregation blurring in the larger census blocks and spatial overglow on the periphery of the most brightly lighted areas. Because both of these unavoidable factors introduce an asymmetric artificial dispersion into the lower tail of the bivariate distribution, we consider the upper tail maximum density to be more representative of the ambient daytime population density. This assumption is consistent with our strategy of varying the low luminance threshold to simulate the effect of increasing connectivity by attenuating the lower luminance pixels that are more likely to represent both types of artifact.

Daily counts of confirmed COVID19 cases, aggregated at the county level for 3262 counties in the continuous USA, were obtained from the Johns Hopkins University of Medicine Coronavirus Resource Center GitHub site (https://github.com/CSSEGISandData/COVID-19). Daily counts of confirmed COVID19 cases, aggregated at the city level for 339 city-specific polygons in China, were obtained from the Harvard Dataverse China Data Lab (https://dataverse.harvard.edu/dataset.xhtml?persistentId=doi:10.7910/DVN/MR5IJN). The construction of the China dataset is described by Hu et al. (2020). Spatially explicit quantities derived from these geocoded time series were gridded in equal area projections (China: Sinusoidal, USA: Molleweide) at 500 m resolution to coregister with the reprojected VIIRS grids. Cumulative confirmed case time series span the period from 1/22/2020 to 8/30/2020 for the US counties and from 1/15/2020 to 8/30/2020 for the China cities.

## Methods

The process of segmenting a continuous luminance field into discrete spatially contiguous subsets of pixels (segments) produces a spatial network in which each segment represents a distinct network component (subset of linked nodes). In each component, adjacent lighted pixels are analogous to nodes with links implied by adjacency. Hence, the spatial networks we consider are defined as sets of network components representing spatially contiguous areas of stable anthropogenic luminance exceeding the specified low luminance threshold. In order to calculate the area of each contiguous network component the 2015 composite is segmented with Queen’s case adjacency neighborhood using each of the three low luminance thresholds and a 9 pixel minimum segment size. For each spatially contiguous segment, area and perimeter are calculated and sorted to produce rank-size distributions. Because segment areas (and perimeters not shown) span 4+ orders of magnitude, the segment area composites use Log_10_ area.

To quantify the scaling properties of the networks, we estimate two parameters of the rank-size distributions: slope (1/(α − 1)) and linear domain. Previous analyses of city population size distributions (Rosen and Resnick 1980; Soo 2005) and other purported power laws (Clauset et al. 2009; Newman 2005) indicate that the estimate of the exponent can be sensitive to biases inherent in the method of estimation. In this study we use the Maximum Likelihood Estimate (MLE) for a power law exponent (α) to quantify the distribution of segment sizes. Although the Ordinary Least Squares (OLS) approach is, by far, the more commonly used method for estimation of Zipf exponents, it suffers from a number of shortcomings as a means of quantifying and testing the power law hypothesis (Clauset et al. 2009; Newman 2005; Sornette 2003). We use more statistically sound estimates of the power law exponent and optimal upper tail cutoff, derived using the MLE and semi-parametric bootstrap approach given by Clauset et al. (2009). The linear domain given by the optimal upper tail cutoff corresponds to the descending rank at which the Maximum Likelihood misfit achieves a minimum and begins to increase.

The spatiotemporal characterization of city (China) and county (USA) level daily confirmed cases of COVID19 follows the methodology described by Small (2012). The Principal Component transformation is applied to the Log_10_ of daily confirmed case time series separately to produce distinct temporal feature spaces for the China city and USA county data. The temporal feature spaces of the spatial principal component loadings show the relationships between the two primary temporal patterns of confirmed case trajectories for each country. Geographic variations in temporal trajectories are manifest by the topology of the temporal feature space. Similarly, the spatial principal component loadings can be projected onto the night light-derived network to illustrate the geographic distributions of different temporal trajectories.

Administrative area choropleth maps of confirmed cases per county are projected onto the intermediate threshold night light network for visualization of the cumulative confirmed cases in the context of the spatial networks of development. Cumulative confirmed cases were gridded using the administrative area shapefile polygons provided with each dataset. Color lookup tables are applied to the choropleth maps to depict increasing numbers of cases along a visible color spectrum with warmer colors corresponding to more cases. These administrative level choropleth maps are projected onto the night light network using a Hue Saturation Value (HSV) color transform of the color-coded choropleth map in which the Value channel is replaced with the normalized luminance value from the night light network then inverse transformed from HSV to RGB. The result is a color map retaining the spatial structure of the night light network, with hue corresponding to the number of confirmed cases and the brightness (Value) corresponding to the luminance as a proxy for the ambient population density. In this representation, more densely populated parts of the network stand out in higher contrast against the black background of unlighted (presumed unpopulated) areas. The first two spatial principal components for the USA confirmed case time series are projected onto the network using the same procedure. In order to calculate the number of confirmed cases within each network component, the total number of cases within each county is normalized by the total area of network components within each county so that total cases summed within components agree with the original county-level case numbers.

## Results

**Fig. 4:**
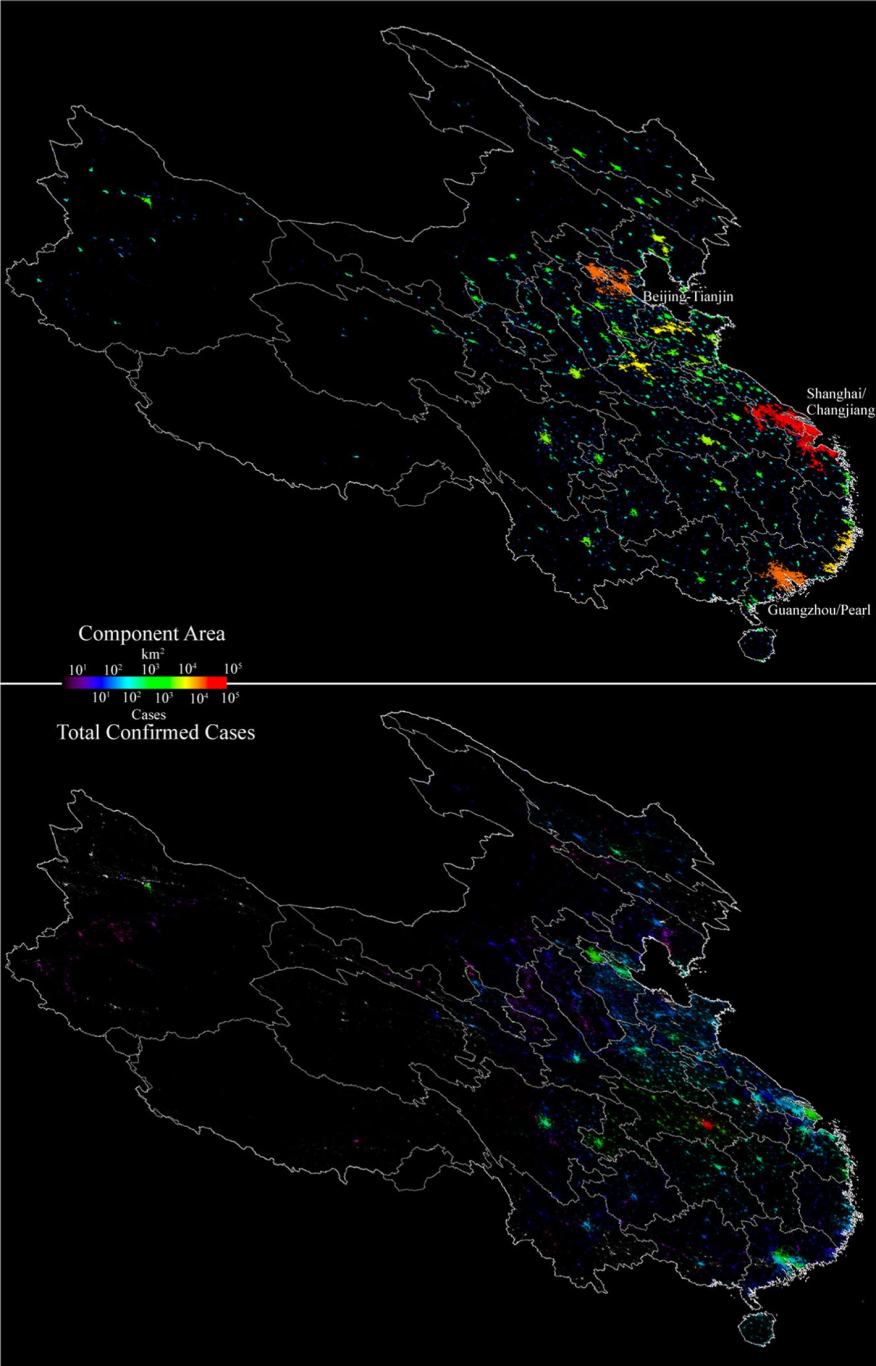
Network component area map and total confirmed case distribution for China. At this scale, the large number of smaller components with fewer cases are not resolved. As expected, the largest number of cases is in Wuhan, while the surrounding smaller components in Hubei have comparable numbers of cases to the high density cores of the much larger components. Enlarge to see detail

**Fig. 5:**
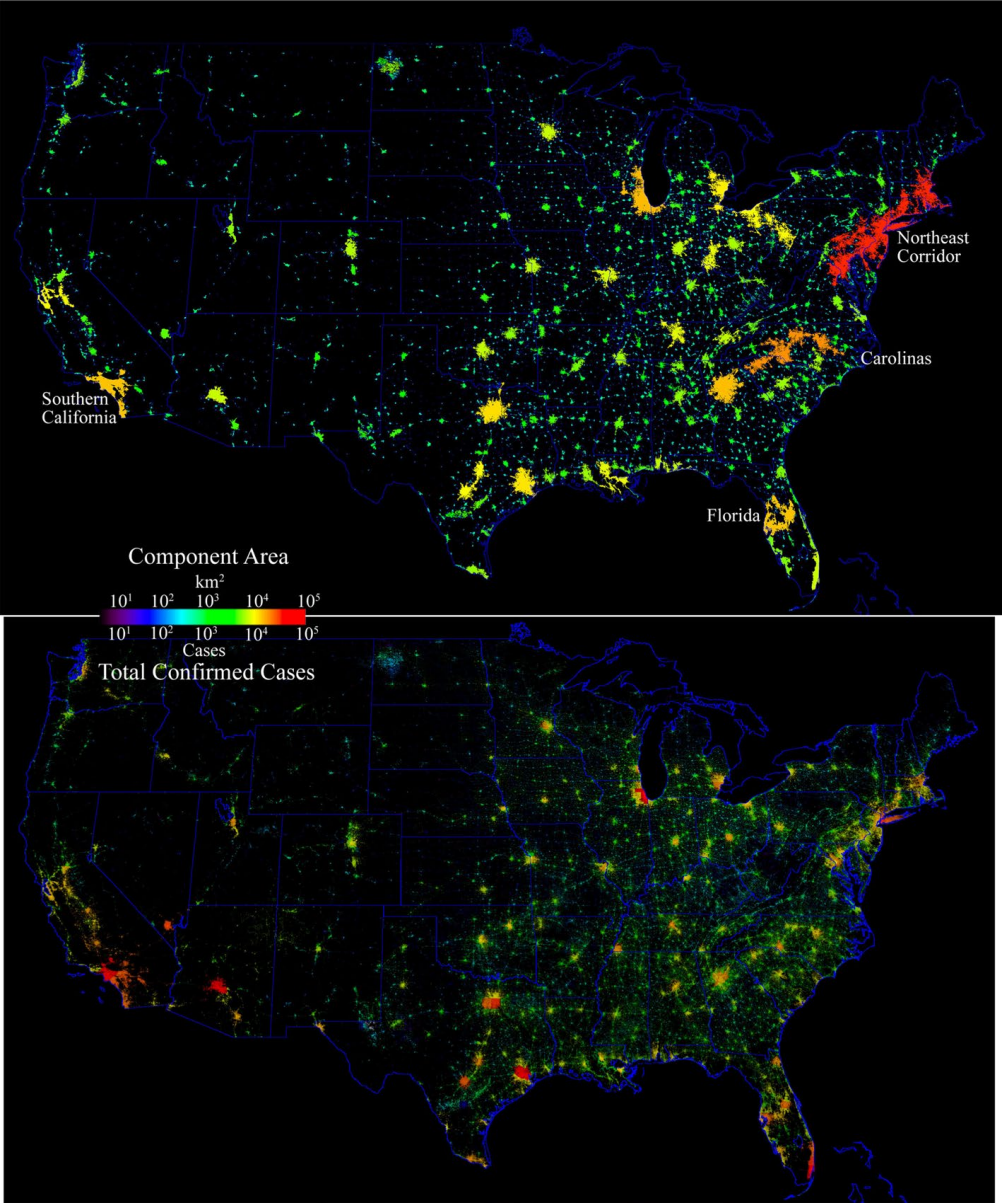
Network component area map and county level confirmed case distribution for the USA. At this scale, the large number of smaller components with fewer cases are not resolved. Case density scales with population density, with the largest numbers in dense urban cores throughout the country. In contrast to China, there is no single epicenter but many large cities with > 10^4^ cases. Similar to China, there are case density gradients within the larger network components. Enlarge to see detail

**Fig. 6:**
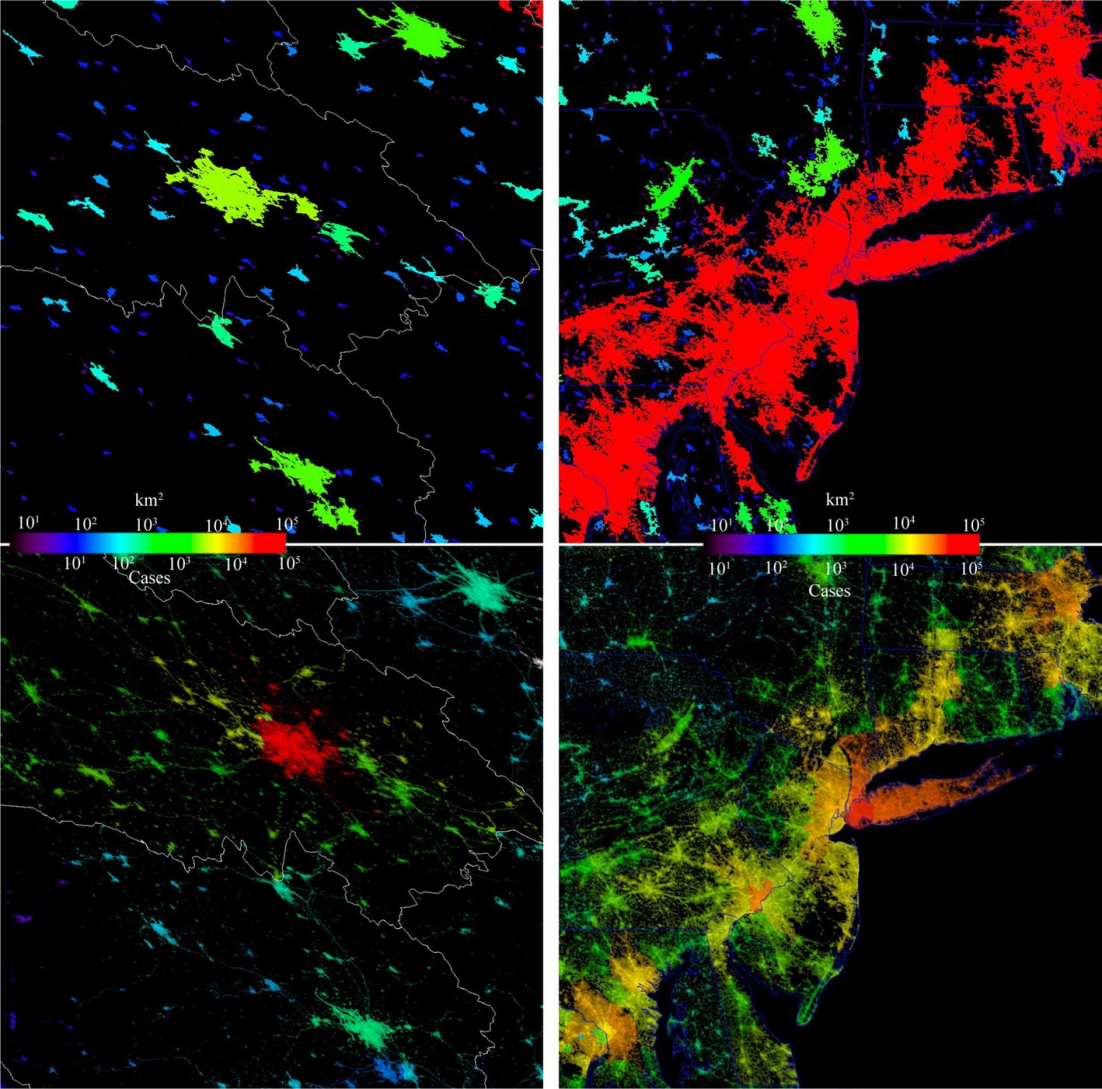
Full resolution comparison of network component area and total confirmed case distributions for Wuhan (left) and the US Northeast Corridor (right). Despite very different network configurations, both examples show spatial gradients in number of confirmed cases with distance from a central hotspot (Wuhan & NYC), however the much larger Northeast Corridor shows strong gradients and multiple local maxima within a single connected component while the smaller components surrounding Wuhan show much smaller numbers of cases. While the Northeast Corridor component is large relative to the spatial gradient in cases, the Wuhan component is much smaller and comparable to the scale of the gradient observed in the NYC hotspot. The distributions are also a result of the disparity in spatial resolution between the 700 m VIIRS resolution and the widely varying sizes of county administrative units within which case counts are aggregated. Each area 500 × 500 km

At national scales, the network component area maps for China and the USA clearly illustrate the characteristic feature of networks of population and development; heavy-tailed distributions containing small numbers of large components and large numbers of small components (Small et al. 2011; Small and Sousa 2016). At the national scale and intermediate luminance threshold shown in Figs. 4 and 5, the large numbers of smaller components are not visible. However, the full resolution illustrations in Fig. 6 show them clearly. Each country’s spatial network shows a continuum of size and number of components, with the largest components generally containing the largest cities.

The cumulative confirmed case distribution maps shown in the lower panels of Figs. 4 and 5 clearly show a stark contrast between the patterns observed in China and the USA. In China, Wuhan stands out as an isolated component with an order of magnitude more cases than the numerous smaller components surrounding it. A radial geographic gradient is apparent, with numbers decreasing with distance before increasing in the urban cores of several larger components. In contrast, the USA has several network components with > 10^4^ confirmed cases in urban cores. Within the larger network components in both China and the USA, conspicuous gradients are apparent, revealing the influence of population density on cumulative case count.

**Fig. 7:**
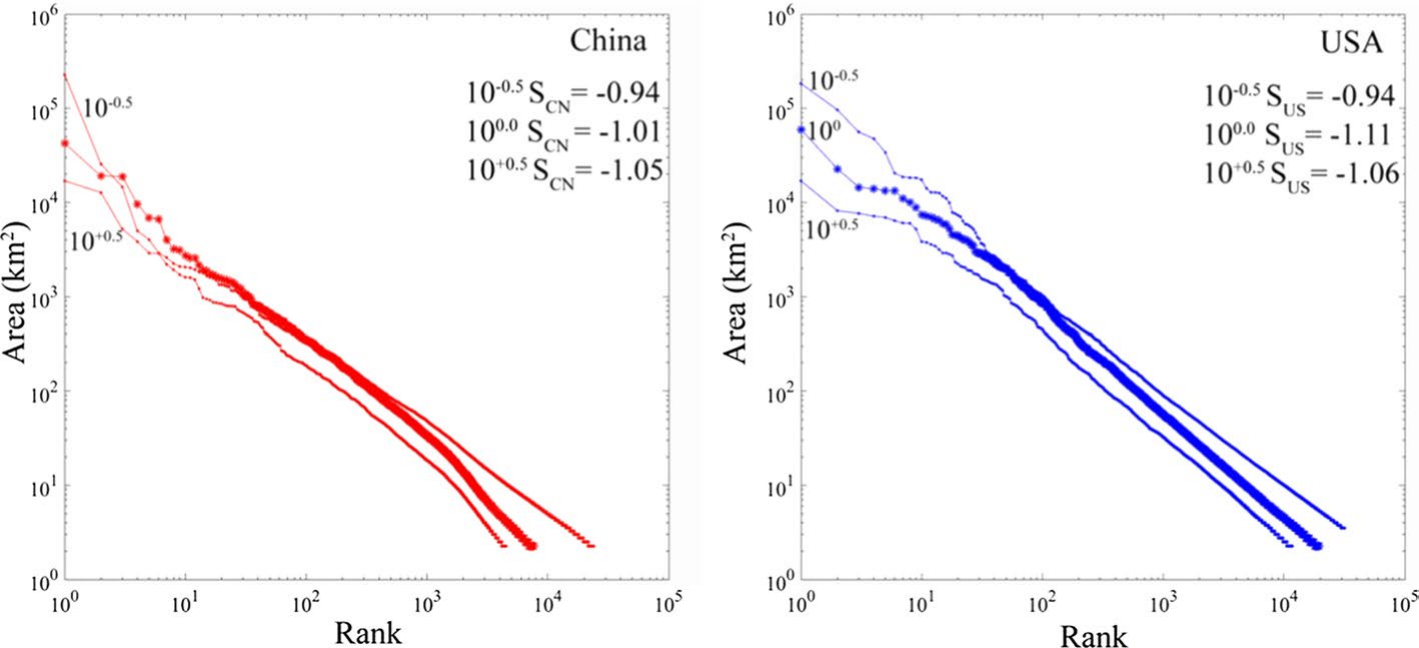
Varying spatial connectivity affects network size, with little effect on scaling (slope). Increasing the low luminance threshold reduces the size, number and connectivity of components, but does not change the slope of the rank-size distributions. For China, the lower threshold results in the interconnection of several large components to form a single very large component on the Changjiang delta and Yangtze River basin—thereby disrupting the scaling of the upper tail. A similar effect is seen in ranks 30 to 100 for the USA

Rank-size plots of component area for the three low luminance thresholds illustrate the effect of varying spatial connectivity on network structure. For both China and the USA, increasing the threshold has the expected effect of reducing number, size and connectivity of network components—as expected (Fig. 7). However, the best fit power law exponents remain near -2, corresponding to rank-size slopes near -1, for all thresholds in both countries. Although numbers and sizes of network components vary by almost an order of magnitude over the range of thresholds, the slopes are relatively invariant, indicating that the scaling properties of both networks are not sensitive to the choice of threshold. The most conspicuous changes are apparent in the uppermost tail of largest components—particularly with the lowest threshold in each country. This is expected because the lowest threshold allows for the most interconnection among large adjacent components, thereby creating a very much larger component in the case of China as the Shanghai component connects to multiple others on the Changjiang Delta and Yangtze River valley. Despite the consistency with a power law spanning 4+ orders of magnitude, we use the exponent estimates only to derive the slope of the rank-size distribution to confirm the uniform area scaling relationship suggesting a dynamic equilibrium between the processes of nucleation, growth and interconnection (Small and Sousa 2016). We emphasize the point made by Broido and Clauset (2019) that it can be very difficult to distinguish true power law scaling from other heavy tailed distributions. Hence, none of our conclusions depend on the scaling being specifically power law.

**Fig. 8:**
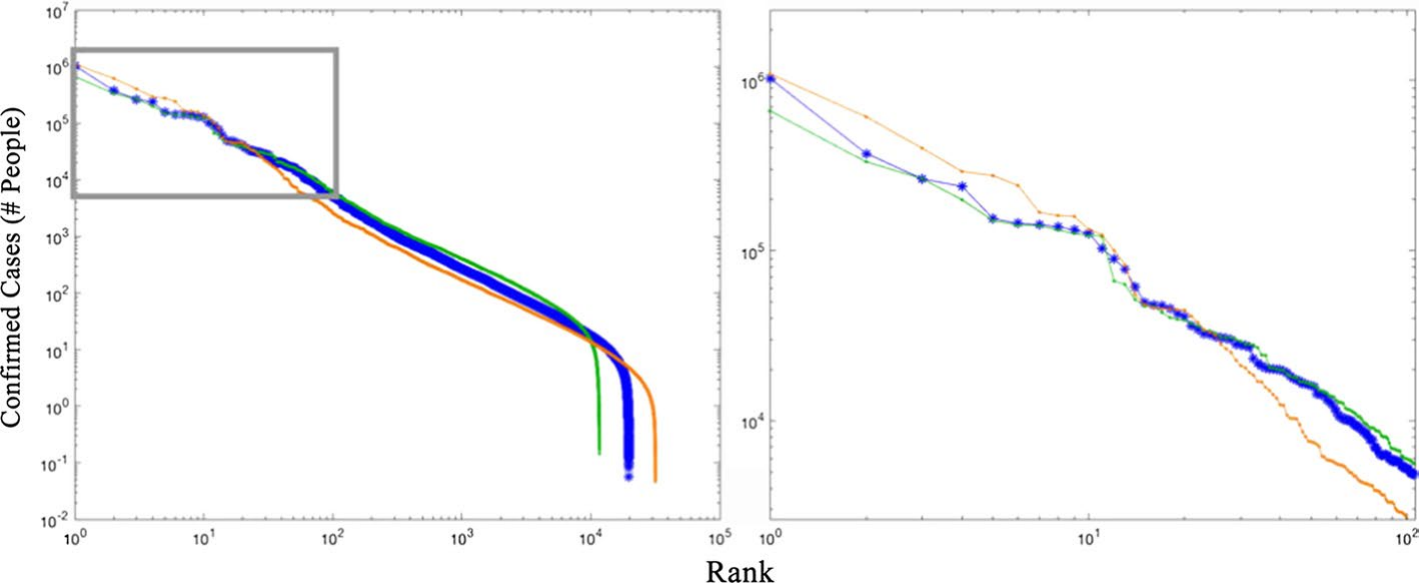
USA COVID-19 confirmed case network scales similarly regardless of low luminance threshold. High threshold (thin green) results in fewer, larger components than low threshold (thin orange). Intermediate threshold (thick blue) shows the emergence of the largest component (Northeast Corridor) but similar scaling to high threshold for other components in the upper tail. All thresholds show rank-size linearity suggesting scale-free behavior spanning 4+ orders of magnitude. Upper tail (gray box; enlarged at right) shows percolation-like behavior at low threshold, with the ~ 30 largest components disproportionately larger than the remainder of the distribution

The much greater spatial resolution of the USA county data than the China city data clearly shows the presence of case density gradients within the larger network components. To quantify the effect of these intra-component gradients, we calculate the total number of confirmed cases within each network component for each low luminance threshold network for the USA. Figure 8 shows rank-size distributions of total cases per component for all three thresholds. The effect of increasing connectivity with decreasing low luminance threshold is seen in the simultaneous growth of the upper tail and shrinkage of the lower tail. This is a result of the presence of high population density urban cores in the largest components contributing disproportionate numbers of cases and introducing discontinuities between the upper and lower tails. This is seen most clearly with the lowest threshold (red) distribution as an offset between the ~ 30 components with the largest number of cases, and the remainder of the distribution.

**Fig. 9:**
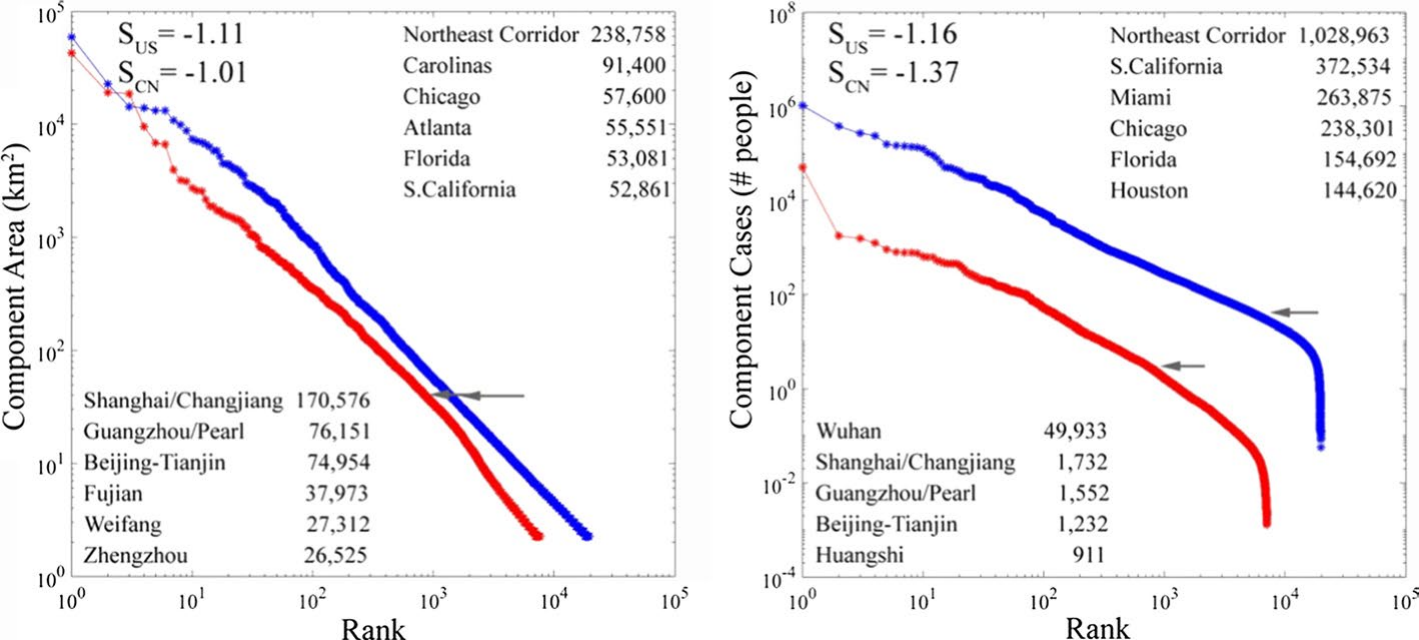
Rank-size distributions for network component areas and cases. As expected, both China and USA show a near-unity slope scaling of component area and rank, indicating a scale-free tradeoff in component number and area. In contrast, the distributions of component case total and rank are steeper, indicating the relatively greater contribution of larger components with higher density cores and lower contribution of small isolated components. Wuhan has disproportionally more cases than the largest components for obvious reasons, although the low numbers of cases of these largest components fall well below the number expected if the scaling were linear as it is in the USA

Direct comparison of the intermediate threshold rank-size distributions of both component area and total number of area-adjusted cumulative case totals shows very similar scaling between China and the USA (Fig. 9). The most conspicuous difference is the dominance of Wuhan in China’s component confirmed case distribution. Aside from Wuhan’s dominance, the confirmed case totals mirror the component areas for China’s network, while the order is different between areas and case totals for the USA network.

**Fig. 10:**
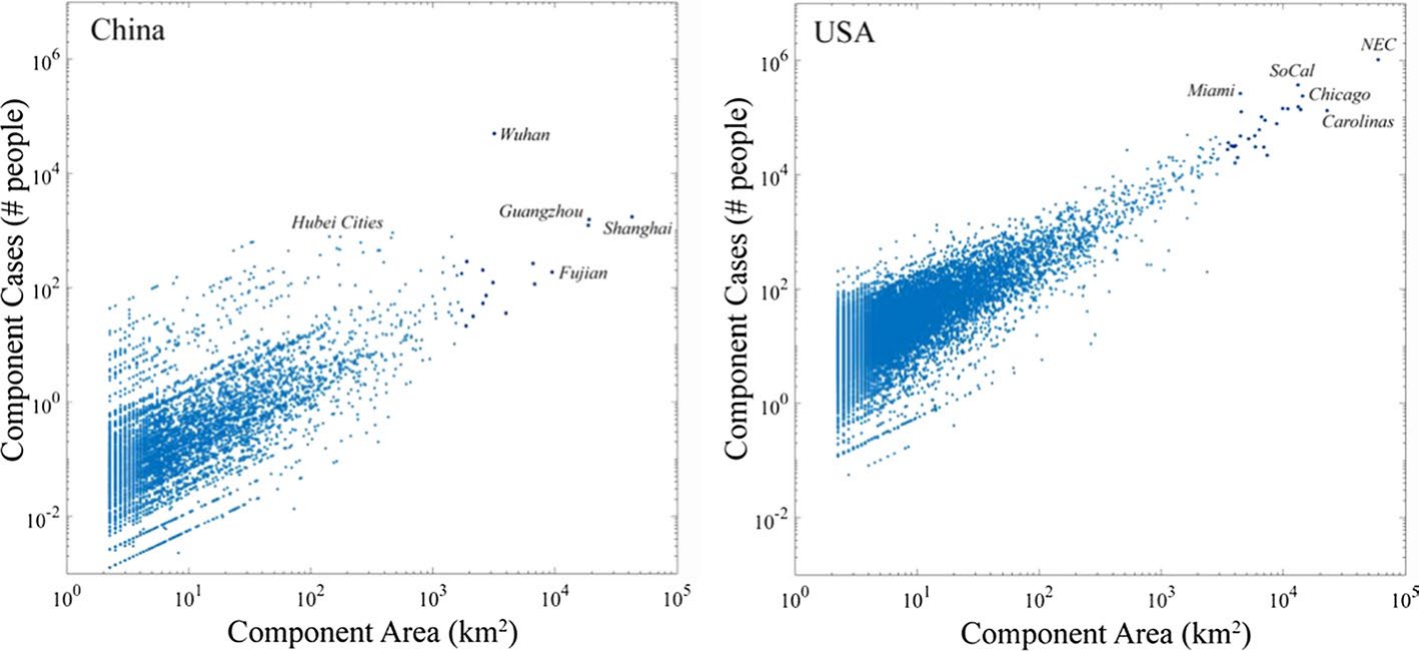
Component case totals versus component areas for China and the USA. The regional scale gradient in case distribution in China results in a wider range, and generally lower number, of cases relative to component area compared to USA. The wide range of cases in smaller components in the USA is a result of small components within larger counties with different numbers of cases. As components become larger than individual counties, this effect is reduced and a consistent trend emerges. Nonetheless, even for large components, considerable variability persists because of both intra-component case density gradients and because of different temporal trajectories leading to differences in total number of cases

Component-specific comparison of component area and case total reveal a key limitation of using spatially aggregated observations. The disparity between the widely varying administrative unit areas, in both China and the USA, and the more detailed and uniform sampling of the VIIRS sensor. Figure 10 shows the expected increase in number of confirmed cases with component area, but also shows a wide range of cases for all but the largest components (of which there are many fewer). While this is partly a result of the geographic gradient in cases with distance from Wuhan in China, a similar pattern of decreasing dispersion with increasing component area is observed in the USA. In part, this reflects the transition from many small components within larger individual counties to fewer larger components encompassing multiple (sometimes many) counties. This artifact of the varying administrative unit area and the aggregation process is an illustration of the Modifiable Areal Unit Problem (https://en.wikipedia.org/wiki/Modifiable_areal_unit_problem) that is unavoidable when using observations aggregated to variable size and shape domains. However, as the range of cases shrinks relative to component area going to larger components encompassing multiple counties, some variation in cases within similarly sized components is still apparent. This illustrates the importance of different temporal trajectories in different regions (network components) of the USA.

**Fig. 11:**
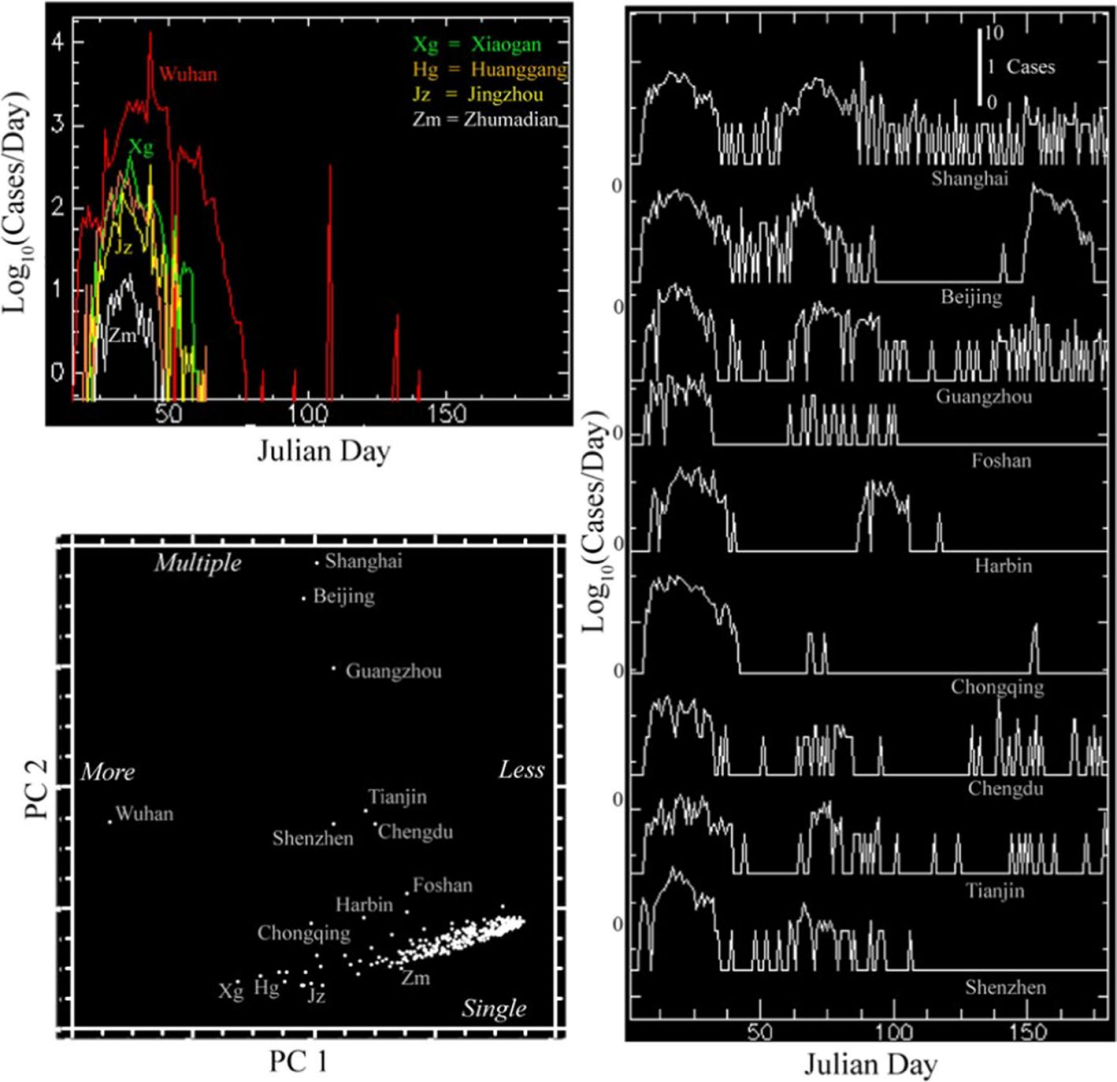
Spatiotemporal analysis of case trajectories in China. The first Principal Component (PC1) clearly corresponds to total number of cases, while the second (PC2) distinguishes larger cities with multiple waves of cases lasting far longer than those observed for Wuhan and other cities in Hubei—which had much larger numbers of cases, albeit in single waves lasting 50 to 75 days

**Fig. 12:**
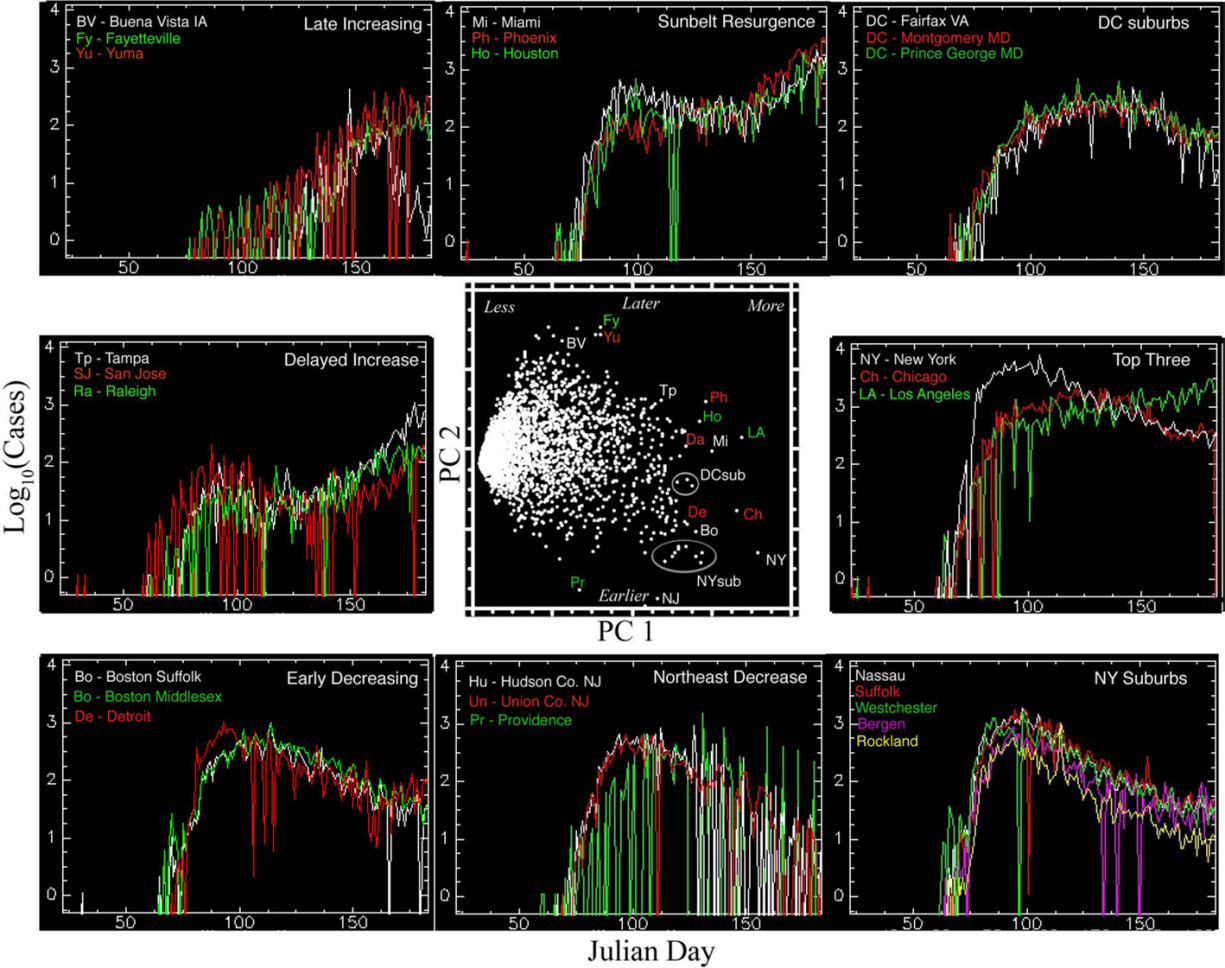
Spatiotemporal analysis of case trajectories in the USA. The first Principal Component also corresponds to total number of cases, while the second corresponds to timing and trend—distinguishing rapid early onset, later decreasing counties from slower, later onset, continued increasing counties

The distributions of temporal trajectories of confirmed cases are very different between China and the USA. While the primary principal component of each corresponds to the total number of cases, the second principal components differ considerably. China shows a strong gradient in total number of cases decreasing with distance from Wuhan in PC1 with an orthogonal limb of 8 large cities showing multiple distinct waves of infections/detections in PC2 (Fig. 11). In contrast, the temporal feature space of the US shows a continuum of total number of cases in PC1 and timing of onset and recovery in PC2 (Fig. 12). The example time series shown in Figs. 11 and 12 illustrate the stark difference between temporal trajectories in China and the USA. Whereas China shows a distinct difference between a few large cities with multiple waves and many cities with single waves of varying duration, the USA shows a much wider variety of temporal trajectories with varying dates and rates of onset and recovery. The first two principal components of the China and USA time series comprise 57% and 65% of their respective total variance, suggesting considerable heterogeneity of trajectories in both countries.

**Fig. 13:**
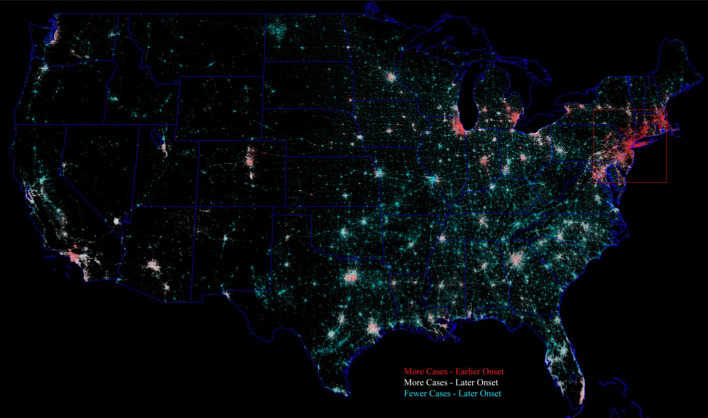
Geographic distribution of number and timing of confirmed cases of COVID-19 for the USA. Principal component loadings from Fig. 12 projected onto spatial network of lighted development show stark contrast between the most distinct temporal trajectories. The Northeast Corridor and Great Lakes Industrial Belt components contrast strongly with larger network components in the south and west and smaller components throughout the country. Note the gradient in case number and timing within the Northeast Corridor component (red box)

The geographic nature of the spatiotemporal evolution of confirmed cases in China is captured by the map of total cases shown in Fig. 4, but the variation in timing and number of cases in the USA is more complex than the map of total cases shown in Fig. 5. The geographic variation in number and timing of cases in the USA is shown by projecting PC1 and PC2 onto the spatial network and depicting their relative contributions with color. Figure 13 illustrates the contrast between network component size and geography with (1) the early onset and recovery of the high density urban cores in the Northeast Corridor and Great Lakes regions, (2) the later onset and sustained increase in the larger cities in the south and west of the country and (3) the consistently lower number of cases in counties with more dispersed populations in larger numbers of smaller network components.

## Discussion

The similarity of the unity slope network scaling in both China and the USA is consistent with continental to global scaling of night light networks (Small 2020; Small et al. 2011)—even when compared for two strongly contrasting economies and cultures at sub-continental scales. The invariance of spatial network scaling to low luminance threshold for both China and the USA further suggests that the network structure is not sensitive to the size of the network or degree of spatial connectivity for networks spanning almost an order of magnitude difference in size and number of components. The implication is that the spatial network depiction of population distribution on the landscape may offer some degree of predictability of future network growth characteristics.

Despite the similarity of network scaling properties in China and the USA, the geographic distributions and spatiotemporal trajectories of confirmed cases suggest very different transmission/detection dynamics. In China the regional localization within medium sized components near Wuhan and teleconnection to larger network components where transmission/detection appears to have been largely contained within the high density urban cores. In contrast, in the USA pervasive propagation throughout the network before mobility restrictions were in place appears to have resulted in a much wider range of temporal trajectories of transmission/detection. This is supported by comparisons of network propagation rates with mobility reduction observations in March 2020 (Small et al. 2020). The conspicuously lower numbers of confirmed cases in China, compared to the USA, is consistent with either underreporting and/or more effective containment. While we are not in a position to speculate about the veracity of the confirmed case data from China, we do note that underreporting and effective containment are not mutually exclusive.

We acknowledge a fundamental ambiguity in the interpretation of confirmed case counts. Absent reliable data on testing frequency in space and time, it is not known to what degree confirmed case data represent disease transmission or testing frequency. This ambiguity can be seen as an example of a phenomenon which could occur in any scenario involving the dispersion of an entity within a spatial network. That is, the ambiguity between the *propagation of the entity itself* (e.g. SARS-CoV-2 pathogen) or the *propagation of the detection effort* (e.g. COVID-19 testing). Spatial observations of propagation within a network can be considered a spatiotemporal convolution of two functions representing (1) propagation of the entity and (2) propagation of detection efforts. While the propagation of confirmed cases through the network is consistent with transmission driven by spatial proximity and population density, it is also conceivable that dense urban cores in larger network components could be the focus of earlier testing efforts which only later may have propagated to smaller, more isolated, less densely populated components.

The use of the spatial network depiction for modeling transmission is clearly only valid for the phase of the pandemic after mobility reductions occurred. The date of initial onset of case increases clearly show that in the USA transmission had already occurred to many of the largest network components by mid-March 2020 when mobility began to decrease in much of the country (Small et al. 2020). Time-lapse cartography of the spatiotemporal evolution of the daily confirmed case counts clearly depicts the slow growth of cases in a small number of network components on the west coast, followed by the appearance of isolated clusters in Phoenix, Chicago and Boston, with an abrupt jump to several other network components in the eastern US in the second week of March. A daily animation is available at: https://www.youtube.com/watch?v=DA-U5XCqLO4.

Mapping confirmed COVID-19 case counts onto the night light-derived map provides a framework for understanding epidemic flow through the human settlement network. A number of interesting features emerge from this analysis. Figures 7 and 8 illustrate two such features, both of which are consistent with previous non-epidemic spatial network analyses. First, increasing/decreasing the low luminance threshold shrinks/grows the network, *but the spatial scaling remains remarkably invariant, retaining its power law-like form*. That being said, at low threshold the ~ 30 segments with the largest number of cases in Fig. 8 begin to exhibit percolation-like behavior, accommodating a disproportionately large fraction of cases relative to the remainder of the distribution. Second, even though segments split/join when the threshold is raised/lowered, *the largest segments generally retain their place near the upper tail of the distribution*. These observations further underscore the important of large conurbations in epidemic spread, as disproportionate numbers of humans are impacted by the epidemic, even relative to their higher population densities. This is consistent with epidemiological expectations due to greater crowding and more frequent rare individuals from the tails of behavior distributions (e.g. superspreaders).

Close examination of the fused night light + COVID-19 network yields additional insights into the structure of the epidemic. For instance, as shown in Figs. 6 and 13, the northeastern US was impacted particularly early and intensely by COVID-19. The major population centers of Boston, New York, Philadelphia, and Baltimore/Washington DC all showed relatively large case counts early in the epidemic. This illustrates two more general features of the analysis framework. First, all of these cities are connected by major highways and commercial development alongside those highways. The bright, spatially extensive lighting associated with these features results in connectivity in the night light image, capturing a real feature of human settlement, mobility and flow which is clearly important in the context of epidemic modeling and control. Second, several major air transit hubs are present in the northeast corridor alone, and at least one major airport is present in each of the segments in the upper tail of the global distribution. Obviously, spatial connectivity of a night light network cannot capture air transit. However, the colocation of major airports and major population centers means that at least some of this information is implicitly captured by the analysis framework. Furthermore, the analysis framework is sufficiently flexible that future studies could explicitly add airport location and activity as a weighting function to investigate the improvement in model behavior.

Several potentially informative avenues exist for future work. A number of additional socioeconomic drivers of health (demographics, income, etc.) could be incorporated as additional spatially explicit network layers. Mobility information from cell networks could be further incorporated to provide spatial constraints on location-specific changes in human behavior. In some instances, temporally variable information can be extracted from the night light signal—with important caveats regarding the potential for challenges due to atmospheric and sensor artifacts.

Finally, appropriate interpretation of the capabilities and limitations of this type of analysis requires an appreciation for the fundamental scale of the analysis. In some instances, spatially targeted analyses with detailed ancillary data may provide localized, neighborhood-level information—but these are the exception. The fixed spatial resolution of the VIIRS sensor is beneficial in providing information with the same level of detail everywhere on Earth—but is also a fundamental limit on the level of detail that can be achieved in complex urban environments. In our opinion, the data used here are best used for understanding epidemic flow at regional to continental scales. Such macroscale analysis is important in its own right for pragmatic purposes, such as resource allocation, logistics, and preparation, as well as theoretical understanding. But we caution against interpretation of these data—or any remotely sensed data—at the pixel level unless additional contextual information is available. That being said, the spatial network framework we propose appears to be scale invariant. It is possible that future sensing capabilities will significantly exceed those of VIIRS and allow for inference at much finer spatial scales. If and when such data are available, the conceptual and analytic approach used here could easily be applied for finer scale analyses.

## Data Availability

All data used in this study are publicly available from the following sources. VIIRS night light composite—https://payneinstitute.mines.edu/eog-2/viirs/ USA county daily COVID-19 confirmed cases: Johns Hopkins University of Medicine Coronavirus Resource Center https://github.com/CSSEGISandData/COVID-19. China city daily counts of confirmed COVID19 cases: Harvard Dataverse China Data Lab https://dataverse.harvard.edu/dataset.xhtml?persistentId=doi:10.7910/DVN/MR5IJN

## Abbreviations

DNB: Day-night band
HSV: Hue Saturation Value
km: Kilometer
m: Meter
nW/(cm^2^ sr): NanoWatts/(centimeter^2^ steradian)
NASA: National Aeronautics and Space Administration
NOAA: National Oceanographic and Atmospheric Administration
NPP: National Polar-orbiting Partnership
PC: Principal Component
RGB: Red Green Blue
USA: United States of America
VIIRS: Visible Infrared Imaging Radiometer Suite
SARS: Severe Acute Respiratory Syndrome
